# First Principles of Medicine

**Published:** 1851-05

**Authors:** 


Art. VII.—*First Principles of Medicine.* By Archibald Billing, M. D., A. M., F. R. S. Second American, from the revised and improved fifth London Edition. Philadelphia: Lea & Blanchard. 1851.

This work is well adapted to two classes of medical men, which embrace nearly the whole profession—those who have a long and familiar acquaintance with the diseased actions of the human economy and the use of therapeutic agents, and those who are desirous of making acquaintance with these interesting phenomena. The first may find a solution of many mysterious occurrences, whose nature they could not understand at the time they happened; the last may know their meaning as they spring up.

Dr. Billing's work can scarcely be regarded as a systematic work on practice, and the routinist who loves to turn to a particular disease to find out what to give, would leave this book in despair. But the physician who wishes to know the philosophy of disease and of cure, may study these pages with no ordinary degree of satisfaction, and he will assuredly rise from the perusal of the work with his confidence in the therapeutical agents of his profession greatly enlarged. The merits of the work may be considered as well established, in the fact that five English editions and two American editions have been published. The student who may devote himself to the work will be at no loss to understand why the book has manifested these signs of popularity, and why the profession has exhibited such an estimate of its value. Almost every page is replete with practical knowledge, and the intelligent practitioner may find here a clue that will lead him through devious labyrinths.

We are far from coinciding in all the views advocated by Dr. Billing. Philosophic truth is not always attained in the dashing, off-hand, *ad captandem* style used too frequently by our author.

It would be a difficult thing to give a correct idea of this work by anything like a synopsis, or by any series of quotations. There is scarcely any item in it that is not dependent upon other portions of the book, and we can give no better advice to our readers than to urge them to read the work carefully and study it thoroughly. They will find themselves abundantly compensated for the labor, if labor they find the pleasures connected with a large increase of knowledge.

We regretted to see a valuable work like this, published by as respectable a house as that of Lea & Blanchard, disfigured from the beginning to the end with typographical blunders. They occur so frequently in some of the forms that we were tempted to believe that the proof-sheets had never been read. The printers of this edition, at West Brookfield, Mass., have shown a lamentable degree of negligence in their part of this publication, that will not enhance the character of their establishment. At any rate, they will not soon rival the fame of the Stephens, nor of Caddell. B.

